# {2,2′-[Ethyl­enebis(nitrilo­methyl­idyne)]diphenolato-κ^4^ *O*,*N*,*N*′,*O*′}oxido­vanadium(IV)

**DOI:** 10.1107/S1600536808010611

**Published:** 2008-05-07

**Authors:** Cheng Wang, Ji-Hong Yuan, Gang Xie, Ming-Juan Yu, Jing Li

**Affiliations:** a School of Chemistry and Materials Science, Heilongjiang University, Harbin 150080, People’s Republic of China; b Mudanjiang Lingtai Pharmaceutical Co. Ltd, Heilongjiang University, Mudanjiang 157000, People’s Republic of China

## Abstract

The title compound, [V(C_16_H_14_N_2_O_2_)O], was synthesized by the reaction of vanadyl(IV) sulfate and *N*,*N*′-bis­(salicyl­idene)ethyl­enediamine under hydro­thermal conditions. The asymmetric unit contains two mol­ecules. Each V^IV^ atom is coordinated in a square-pyramidal geometry by two N atoms and two O atoms from a ligand in the basal plane and by an oxide O atom in the apical position. Weak C—H⋯O hydrogen bonds lead to a three-dimensional supra­molecular structure.

## Related literature

For related literature, see: Butler & Walker (1993 ▶); Deng *et al.* (2007 ▶); Martinez *et al.* (2001 ▶); Sun *et al.* (1996 ▶).
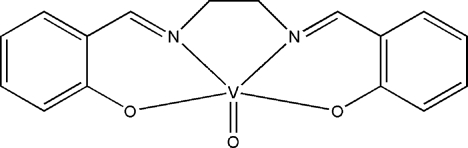


## Experimental

### 

#### Crystal data

[V(C_16_H_14_N_2_O_2_)O]
*M* *_r_* = 333.23
Monoclinic, 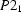

*a* = 13.648 (3) Å
*b* = 6.8085 (14) Å
*c* = 15.952 (3) Å
β = 98.24 (3)°
*V* = 1466.9 (5) Å^3^
*Z* = 4
Mo *K*α radiation
μ = 0.69 mm^−1^
*T* = 293 (2) K
0.32 × 0.21 × 0.11 mm

#### Data collection

Rigaku R-AXIS RAPID diffractometer
Absorption correction: multi-scan (*ABSCOR*; Higashi, 1995 ▶) *T*_min_ = 0.809, *T*_max_ = 0.930
14358 measured reflections
6102 independent reflections
4561 reflections with *I* > 2σ(*I*)
*R*_int_ = 0.029

#### Refinement

*R*[*F* ^2^ > 2σ(*F* ^2^)] = 0.043
*wR*(*F* ^2^) = 0.109
*S* = 1.03
6102 reflections
397 parameters
2 restraints
H-atom parameters constrained
Δρ_max_ = 0.56 e Å^−3^
Δρ_min_ = −0.43 e Å^−3^
Absolute structure: Flack (1983 ▶), 2456 Friedel pairs
Flack parameter: 0.01 (2)

### 

Data collection: *PROCESS-AUTO* (Rigaku, 1998 ▶); cell refinement: *PROCESS-AUTO*; data reduction: *PROCESS-AUTO*; program(s) used to solve structure: *SHELXS97* (Sheldrick, 2008 ▶); program(s) used to refine structure: *SHELXL97* (Sheldrick, 2008 ▶); molecular graphics: *SHELXTL-Plus* (Sheldrick, 2008 ▶); software used to prepare material for publication: *SHELXL97*.

## Supplementary Material

Crystal structure: contains datablocks global, I. DOI: 10.1107/S1600536808010611/hy2124sup1.cif

Structure factors: contains datablocks I. DOI: 10.1107/S1600536808010611/hy2124Isup2.hkl

Additional supplementary materials:  crystallographic information; 3D view; checkCIF report

## Figures and Tables

**Table:** 

V1—O1	1.584 (3)
V1—O4	1.922 (3)
V1—O3	1.931 (2)
V1—N1	2.058 (3)
V1—N2	2.059 (3)
V2—O2	1.582 (3)
V2—O5	1.917 (3)
V2—O6	1.926 (2)
V2—N3	2.056 (3)
V2—N4	2.067 (3)

**Table:** 

O1—V1—O4	111.65 (15)
O1—V1—O3	106.38 (13)
O4—V1—O3	88.68 (10)
O1—V1—N1	107.53 (15)
O4—V1—N1	140.24 (11)
O3—V1—N1	86.73 (11)
O1—V1—N2	102.72 (13)
O4—V1—N2	86.65 (11)
O3—V1—N2	150.17 (11)
N1—V1—N2	78.34 (12)
O2—V2—O5	109.95 (15)
O2—V2—O6	106.92 (15)
O5—V2—O6	88.01 (11)
O2—V2—N3	105.39 (15)
O5—V2—N3	87.24 (12)
O6—V2—N3	147.00 (12)
O2—V2—N4	107.48 (17)
O5—V2—N4	142.21 (13)
O6—V2—N4	86.06 (13)
N3—V2—N4	78.04 (14)

**Table 2:** Hydrogen-bond geometry (Å, °)

*D*—H⋯*A*	*D*—H	H⋯*A*	*D*⋯*A*	*D*—H⋯*A*
C8—H8*B*⋯O4^i^	0.97	2.55	3.139 (3)	119
C14—H14⋯O3^ii^	0.93	2.56	3.364 (3)	145
C24—H24*B*⋯O1^iii^	0.97	2.34	3.178 (3)	144

Symmetry codes: (i) 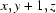; (ii) 
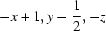
; (iii) 
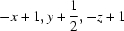
.

**Fig. 1.**
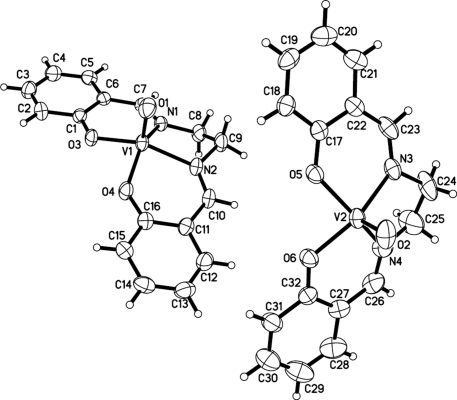
The molecular structure of the title compound. Displacement ellipsoids are drawn at the 30% probability level.

**Fig. 2.**
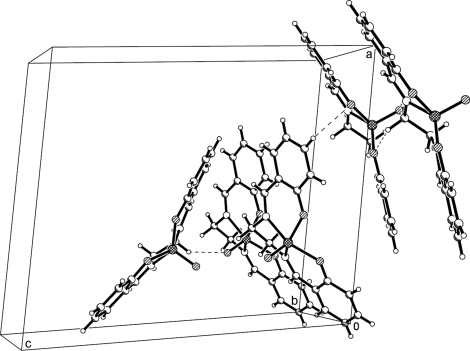
A view of the crystal packing. Hydrogen bonds are drawn as dashed lines.

## supplementary crystallographic information

### Comment

In the past few decades, there has been increased interest in coordination chemistry and biochemistry of oxovanadium. The main reason is that the function of vanadium in biological system has been recognized, such as the regulation of carbohydrate metabolism (Butler & Walker, 1993; Martinez *et al.*, 2001; Sun *et al.*, 1996). Generally, Schiff base is coordinated to vanadium through O and N atoms (Deng *et al.*, 2007), similar to those in the biological systems. Therefore, it is important to intensively study the relationship of syntheses and structural properties of vanadium-schiff base complexes. We report here the crystal structure of the title compound, a vanadium complex with a schiff-base ligand, *N*,*N*'-bis(salicylidene)ethylenediamine (H_2_salen).

In the asymmetric unit, there are two crystallographically independent molecules (Fig. 1). Each V^IV^ atom is coordinated by two N atoms and two O atoms from a salen ligand in the equatorial plane and an oxido O atom in the apical position, resulting in a five-coordinated square-pyramidal geometry (Table 1). The V ?O bond lengths are 1.582 (2) and 1.583 (2) Å, which are in the normal range. In the crystal structure, though each salen ligand contains two phenyl ring with dihedral angles of 156.3 (4)° and 164.1 (4)°, respectively, there are no π–π interactions between the molecules. In virtue of weak intermolecular C—H···O hydrogen bonds (Table 2), a three-dimensional hydrogen-bonding network is constructed (Fig. 2).

### Experimental

A mixture of VOSO_4_.4H_2_O (1.175 g, 5 mmol), H_2_salen (0.725 g, 5 mmol), triethylamine (0.20 mmol) and methanol (0.50 mol) in water was stirred for 1 h, and then heated at 413 K for 3 d in a sealed 25 ml Teflon-lined stainless steel vessel under autogenous pressure. After the reaction mixture was slowly cooled to room temperature at a rate of 5 K h^-1^, black block crystals of the title compound were collected by filtration, washed with distilled water and dried in air.

### Refinement

H atoms were positioned geometrically and refined as riding atoms, with C—H = 0.93 (aromatic), 0.97Å (CH_2_) and *U*_iso_(H) = 1.2*U*_eq_(C).

### Crystal data

**Table:**

[V(C_16_H_14_N_2_O_2_)O]	*F*_000_ = 684
*M**_r_* = 333.23	*D*_x_ = 1.509 Mg m^−^^3^
Monoclinic, *P*2_1_	Mo *K*α radiation λ = 0.71073 Å
Hall symbol: P 2yb	Cell parameters from 9875 reflections
*a* = 13.648 (3) Å	θ = 3.0–27.5º
*b* = 6.8085 (14) Å	µ = 0.69 mm^−^^1^
*c* = 15.952 (3) Å	*T* = 293 (2) K
β = 98.24 (3)º	Block, black
*V* = 1466.9 (5) Å^3^	0.32 × 0.21 × 0.11 mm
*Z* = 4	

### Data collection

**Table:**

Rigaku R-AXIS RAPID diffractometer	6102 independent reflections
Radiation source: rotating anode	4561 reflections with *I* > 2σ(*I*)
Monochromator: graphite	*R*_int_ = 0.029
Detector resolution: 10.0 pixels mm^-1^	θ_max_ = 27.5º
*T* = 293(2) K	θ_min_ = 3.0º
ω scans	*h* = −17→17
Absorption correction: multi-scan(ABSCOR; Higashi, 1995)	*k* = −8→8
*T*_min_ = 0.809, *T*_max_ = 0.930	*l* = −20→19
14358 measured reflections	

### Refinement

**Table:**

Refinement on *F*^2^	Hydrogen site location: inferred from neighbouring sites
Least-squares matrix: full	H-atom parameters constrained
*R*[*F*^2^ > 2σ(*F*^2^)] = 0.043	*w* = 1/[σ^2^(*F*_o_^2^) + (0.0582*P*)^2^ + 0.1081*P*] where *P* = (*F*_o_^2^ + 2*F*_c_^2^)/3
*wR*(*F*^2^) = 0.109	(Δ/σ)_max_ = 0.006
*S* = 1.03	Δρ_max_ = 0.57 e Å^−^^3^
6102 reflections	Δρ_min_ = −0.43 e Å^−^^3^
397 parameters	Extinction correction: none
2 restraints	Absolute structure: Flack (1983), 2456 Friedel pairs
Primary atom site location: structure-invariant direct methods	Flack parameter: 0.01 (2)
Secondary atom site location: difference Fourier map	

### Special details

**Table:**

Geometry. All e.s.d.'s (except the e.s.d. in the dihedral angle between two l.s. planes) are estimated using the full covariance matrix. The cell e.s.d.'s are taken into account individually in the estimation of e.s.d.'s in distances, angles and torsion angles; correlations between e.s.d.'s in cell parameters are only used when they are defined by crystal symmetry. An approximate (isotropic) treatment of cell e.s.d.'s is used for estimating e.s.d.'s involving l.s. planes.

### Fractional atomic coordinates and isotropic or equivalent isotropic displacement parameters (Å^2^)

**Table:**

	*x*	*y*	*z*	*U*_iso_*/*U*_eq_	
V1	0.27998 (4)	0.86758 (8)	0.08474 (3)	0.04713 (15)	
V2	0.73287 (4)	0.88060 (8)	0.57991 (3)	0.04920 (15)	
N1	0.2175 (2)	1.1335 (4)	0.04608 (19)	0.0488 (7)	
N2	0.3847 (2)	1.0619 (4)	0.14130 (17)	0.0510 (7)	
N3	0.6545 (3)	1.1035 (4)	0.62647 (19)	0.0596 (8)	
N4	0.7996 (3)	1.1273 (4)	0.5380 (2)	0.0683 (9)	
O1	0.2286 (2)	0.7938 (4)	0.16192 (18)	0.0759 (8)	
O2	0.8039 (3)	0.7978 (4)	0.65914 (19)	0.0841 (9)	
O3	0.20463 (17)	0.7577 (3)	−0.01603 (15)	0.0507 (6)	
O4	0.38881 (19)	0.7014 (3)	0.06653 (15)	0.0565 (6)	
O5	0.61179 (19)	0.7339 (3)	0.56339 (14)	0.0555 (6)	
O6	0.7688 (2)	0.7514 (4)	0.48130 (16)	0.0598 (6)	
C1	0.1221 (3)	0.8198 (5)	−0.0612 (2)	0.0455 (8)	
C2	0.0694 (3)	0.6938 (6)	−0.1214 (2)	0.0602 (10)	
H2	0.0934	0.5679	−0.1283	0.072*	
C3	−0.0166 (3)	0.7526 (7)	−0.1700 (3)	0.0677 (11)	
H3	−0.0504	0.6651	−0.2086	0.081*	
C4	−0.0541 (3)	0.9403 (6)	−0.1627 (2)	0.0618 (10)	
H4	−0.1125	0.9790	−0.1959	0.074*	
C5	−0.0043 (3)	1.0658 (6)	−0.1062 (2)	0.0565 (9)	
H5	−0.0292	1.1917	−0.1011	0.068*	
C6	0.0835 (3)	1.0116 (5)	−0.0554 (2)	0.0465 (8)	
C7	0.1371 (3)	1.1600 (5)	−0.0042 (2)	0.0508 (8)	
H7	0.1111	1.2865	−0.0079	0.061*	
C8	0.2716 (3)	1.3091 (5)	0.0809 (2)	0.0613 (10)	
H8A	0.2253	1.4092	0.0937	0.074*	
H8B	0.3107	1.3622	0.0401	0.074*	
C9	0.3379 (3)	1.2495 (5)	0.1604 (3)	0.0655 (10)	
H9A	0.3879	1.3491	0.1764	0.079*	
H9B	0.2996	1.2324	0.2067	0.079*	
C10	0.4778 (3)	1.0407 (5)	0.1580 (2)	0.0556 (9)	
H10	0.5143	1.1453	0.1836	0.067*	
C11	0.5313 (3)	0.8654 (7)	0.13993 (19)	0.0540 (7)	
C12	0.6340 (3)	0.8573 (8)	0.1668 (2)	0.0697 (10)	
H12	0.6657	0.9654	0.1942	0.084*	
C13	0.6879 (3)	0.6955 (8)	0.1537 (3)	0.0760 (13)	
H13	0.7557	0.6931	0.1720	0.091*	
C14	0.6415 (3)	0.5350 (7)	0.1133 (2)	0.0702 (12)	
H14	0.6780	0.4227	0.1058	0.084*	
C15	0.5407 (3)	0.5390 (6)	0.0837 (2)	0.0584 (9)	
H15	0.5109	0.4309	0.0549	0.070*	
C16	0.4833 (3)	0.7039 (5)	0.0967 (2)	0.0515 (8)	
C17	0.5307 (3)	0.7501 (5)	0.5992 (2)	0.0506 (8)	
C18	0.4647 (3)	0.5918 (7)	0.5952 (2)	0.0599 (9)	
H18	0.4790	0.4774	0.5675	0.072*	
C19	0.3798 (3)	0.6014 (8)	0.6310 (2)	0.0695 (11)	
H19	0.3383	0.4926	0.6283	0.083*	
C20	0.3545 (3)	0.7698 (8)	0.6711 (3)	0.0798 (13)	
H20	0.2964	0.7748	0.6952	0.096*	
C21	0.4155 (3)	0.9279 (7)	0.6749 (2)	0.0707 (12)	
H21	0.3975	1.0427	0.7004	0.085*	
C22	0.5057 (3)	0.9226 (5)	0.6412 (2)	0.0561 (9)	
C23	0.5682 (4)	1.0928 (6)	0.6496 (2)	0.0658 (11)	
H23	0.5445	1.2043	0.6738	0.079*	
C24	0.7116 (5)	1.2892 (6)	0.6381 (3)	0.0991 (19)	
H24A	0.6669	1.3988	0.6419	0.119*	
H24B	0.7578	1.2838	0.6903	0.119*	
C25	0.7656 (5)	1.3167 (7)	0.5658 (4)	0.119 (2)	
H25A	0.7226	1.3786	0.5196	0.143*	
H25B	0.8220	1.4023	0.5819	0.143*	
C26	0.8629 (4)	1.1322 (6)	0.4880 (3)	0.0761 (12)	
H26	0.8954	1.2507	0.4828	0.091*	
C27	0.8889 (3)	0.9717 (6)	0.4386 (3)	0.0642 (11)	
C28	0.9623 (4)	0.9992 (9)	0.3841 (3)	0.0943 (16)	
H28	0.9960	1.1180	0.3849	0.113*	
C29	0.9831 (4)	0.8558 (12)	0.3318 (3)	0.1094 (19)	
H29	1.0310	0.8764	0.2967	0.131*	
C30	0.9342 (4)	0.6780 (9)	0.3294 (3)	0.0943 (16)	
H30	0.9491	0.5799	0.2927	0.113*	
C31	0.8646 (3)	0.6461 (7)	0.3805 (3)	0.0742 (12)	
H31	0.8327	0.5251	0.3785	0.089*	
C32	0.8391 (3)	0.7910 (6)	0.4364 (2)	0.0571 (9)	

### Atomic displacement parameters (Å^2^)

**Table:**

	*U*^11^	*U*^22^	*U*^33^	*U*^12^	*U*^13^	*U*^23^
V1	0.0570 (3)	0.0332 (2)	0.0502 (3)	−0.0080 (3)	0.0042 (2)	−0.0022 (3)
V2	0.0635 (4)	0.0335 (3)	0.0486 (3)	0.0022 (3)	0.0011 (2)	−0.0011 (3)
N1	0.0523 (18)	0.0317 (13)	0.0634 (17)	−0.0046 (11)	0.0115 (14)	−0.0086 (12)
N2	0.058 (2)	0.0402 (15)	0.0517 (15)	−0.0092 (13)	−0.0015 (14)	−0.0046 (13)
N3	0.086 (3)	0.0376 (15)	0.0562 (17)	−0.0007 (15)	0.0125 (17)	−0.0066 (13)
N4	0.070 (2)	0.0378 (15)	0.099 (3)	−0.0036 (14)	0.021 (2)	−0.0029 (16)
O1	0.091 (2)	0.0718 (18)	0.0661 (16)	−0.0279 (15)	0.0150 (15)	0.0010 (13)
O2	0.107 (2)	0.0601 (16)	0.0731 (17)	0.0166 (16)	−0.0276 (17)	−0.0025 (14)
O3	0.0551 (15)	0.0352 (11)	0.0596 (13)	−0.0047 (10)	0.0002 (11)	−0.0074 (10)
O4	0.0562 (16)	0.0389 (12)	0.0703 (15)	−0.0014 (11)	−0.0049 (12)	−0.0058 (12)
O5	0.0669 (17)	0.0444 (13)	0.0576 (13)	−0.0018 (11)	0.0176 (12)	−0.0068 (11)
O6	0.0676 (17)	0.0475 (14)	0.0673 (15)	−0.0011 (12)	0.0199 (13)	−0.0065 (12)
C1	0.048 (2)	0.0438 (19)	0.0456 (16)	−0.0060 (13)	0.0084 (15)	−0.0008 (13)
C2	0.073 (3)	0.048 (2)	0.058 (2)	−0.0034 (18)	0.004 (2)	−0.0103 (18)
C3	0.064 (3)	0.077 (3)	0.060 (2)	−0.013 (2)	−0.001 (2)	−0.011 (2)
C4	0.054 (2)	0.083 (3)	0.0466 (18)	0.0014 (19)	−0.0004 (16)	0.0002 (18)
C5	0.053 (2)	0.062 (2)	0.0548 (19)	0.0058 (18)	0.0100 (17)	0.0008 (18)
C6	0.048 (2)	0.0452 (18)	0.0482 (17)	−0.0047 (14)	0.0137 (15)	−0.0017 (15)
C7	0.049 (2)	0.0370 (17)	0.068 (2)	0.0027 (14)	0.0119 (18)	0.0022 (15)
C8	0.068 (3)	0.0352 (17)	0.080 (3)	−0.0031 (15)	0.007 (2)	−0.0101 (16)
C9	0.076 (3)	0.0449 (19)	0.073 (2)	−0.0094 (18)	0.003 (2)	−0.0191 (19)
C10	0.066 (3)	0.0445 (19)	0.0522 (19)	−0.0122 (16)	−0.0066 (17)	−0.0051 (16)
C11	0.057 (2)	0.0536 (18)	0.0494 (15)	−0.005 (2)	0.0014 (14)	0.004 (2)
C12	0.062 (2)	0.087 (3)	0.0583 (19)	−0.010 (3)	0.0032 (17)	−0.008 (3)
C13	0.051 (3)	0.115 (4)	0.061 (2)	0.003 (3)	0.0072 (19)	0.002 (3)
C14	0.066 (3)	0.093 (3)	0.054 (2)	0.020 (2)	0.017 (2)	0.005 (2)
C15	0.065 (3)	0.056 (2)	0.0548 (19)	0.0054 (18)	0.0095 (18)	−0.0010 (18)
C16	0.060 (2)	0.0502 (19)	0.0432 (17)	−0.0002 (16)	0.0024 (16)	0.0043 (16)
C17	0.058 (2)	0.0517 (19)	0.0398 (16)	0.0064 (17)	−0.0009 (15)	0.0057 (15)
C18	0.060 (3)	0.066 (2)	0.0493 (19)	−0.0012 (19)	−0.0042 (17)	0.0007 (18)
C19	0.056 (3)	0.090 (3)	0.058 (2)	−0.010 (2)	−0.0083 (19)	0.013 (2)
C20	0.060 (3)	0.112 (4)	0.067 (2)	0.011 (3)	0.010 (2)	0.024 (3)
C21	0.075 (3)	0.077 (3)	0.061 (2)	0.026 (2)	0.015 (2)	0.013 (2)
C22	0.065 (2)	0.058 (2)	0.0454 (16)	0.0140 (17)	0.0061 (16)	0.0068 (16)
C23	0.110 (4)	0.0428 (19)	0.0494 (19)	0.016 (2)	0.026 (2)	0.0037 (17)
C24	0.183 (6)	0.037 (2)	0.085 (3)	−0.026 (3)	0.045 (4)	−0.020 (2)
C25	0.143 (5)	0.051 (3)	0.177 (6)	−0.023 (3)	0.071 (5)	−0.035 (3)
C26	0.073 (3)	0.048 (2)	0.108 (3)	−0.0116 (19)	0.013 (3)	0.010 (2)
C27	0.058 (3)	0.062 (2)	0.073 (3)	0.0045 (19)	0.010 (2)	0.016 (2)
C28	0.087 (4)	0.090 (4)	0.111 (4)	−0.003 (3)	0.032 (3)	0.030 (3)
C29	0.117 (4)	0.123 (5)	0.101 (3)	0.018 (5)	0.058 (3)	0.015 (4)
C30	0.108 (4)	0.100 (4)	0.082 (3)	0.008 (3)	0.039 (3)	0.001 (3)
C31	0.075 (3)	0.076 (3)	0.075 (3)	0.005 (2)	0.023 (2)	−0.008 (2)
C32	0.052 (2)	0.059 (2)	0.058 (2)	0.0048 (17)	0.0010 (18)	0.0065 (17)

### Geometric parameters (Å, °)

**Table:**

V1—O1	1.584 (3)	C10—H10	0.9300
V1—O4	1.922 (3)	C11—C12	1.407 (5)
V1—O3	1.931 (2)	C11—C16	1.408 (5)
V1—N1	2.058 (3)	C12—C13	1.358 (7)
V1—N2	2.059 (3)	C12—H12	0.9300
V2—O2	1.582 (3)	C13—C14	1.376 (7)
V2—O5	1.917 (3)	C13—H13	0.9300
V2—O6	1.926 (2)	C14—C15	1.390 (6)
V2—N3	2.056 (3)	C14—H14	0.9300
V2—N4	2.067 (3)	C15—C16	1.401 (5)
N1—C7	1.275 (4)	C15—H15	0.9300
N1—C8	1.472 (4)	C17—C18	1.401 (5)
N2—C10	1.268 (5)	C17—C22	1.418 (5)
N2—C9	1.480 (5)	C18—C19	1.364 (6)
N3—C23	1.286 (5)	C18—H18	0.9300
N3—C24	1.482 (5)	C19—C20	1.381 (7)
N4—C26	1.258 (5)	C19—H19	0.9300
N4—C25	1.461 (6)	C20—C21	1.357 (7)
O3—C1	1.316 (4)	C20—H20	0.9300
O4—C16	1.311 (4)	C21—C22	1.412 (5)
O5—C17	1.320 (4)	C21—H21	0.9300
O6—C32	1.305 (5)	C22—C23	1.434 (6)
C1—C2	1.406 (5)	C23—H23	0.9300
C1—C6	1.416 (5)	C24—C25	1.468 (6)
C2—C3	1.370 (6)	C24—H24A	0.9700
C2—H2	0.9300	C24—H24B	0.9700
C3—C4	1.388 (6)	C25—H25A	0.9700
C3—H3	0.9300	C25—H25B	0.9700
C4—C5	1.352 (5)	C26—C27	1.422 (6)
C4—H4	0.9300	C26—H26	0.9300
C5—C6	1.396 (5)	C27—C32	1.404 (6)
C5—H5	0.9300	C27—C28	1.429 (6)
C6—C7	1.433 (5)	C28—C29	1.341 (8)
C7—H7	0.9300	C28—H28	0.9300
C8—C9	1.504 (5)	C29—C30	1.380 (9)
C8—H8A	0.9700	C29—H29	0.9300
C8—H8B	0.9700	C30—C31	1.355 (6)
C9—H9A	0.9700	C30—H30	0.9300
C9—H9B	0.9700	C31—C32	1.406 (6)
C10—C11	1.450 (6)	C31—H31	0.9300
			
O1—V1—O4	111.65 (15)	C12—C11—C16	119.4 (4)
O1—V1—O3	106.38 (13)	C12—C11—C10	118.7 (4)
O4—V1—O3	88.68 (10)	C16—C11—C10	121.9 (3)
O1—V1—N1	107.53 (15)	C13—C12—C11	121.5 (5)
O4—V1—N1	140.24 (11)	C13—C12—H12	119.2
O3—V1—N1	86.73 (11)	C11—C12—H12	119.2
O1—V1—N2	102.72 (13)	C12—C13—C14	119.6 (4)
O4—V1—N2	86.65 (11)	C12—C13—H13	120.2
O3—V1—N2	150.17 (11)	C14—C13—H13	120.2
N1—V1—N2	78.34 (12)	C13—C14—C15	120.7 (4)
O2—V2—O5	109.95 (15)	C13—C14—H14	119.7
O2—V2—O6	106.92 (15)	C15—C14—H14	119.7
O5—V2—O6	88.01 (11)	C14—C15—C16	120.8 (4)
O2—V2—N3	105.39 (15)	C14—C15—H15	119.6
O5—V2—N3	87.24 (12)	C16—C15—H15	119.6
O6—V2—N3	147.00 (12)	O4—C16—C15	118.5 (3)
O2—V2—N4	107.48 (17)	O4—C16—C11	123.6 (3)
O5—V2—N4	142.21 (13)	C15—C16—C11	117.9 (3)
O6—V2—N4	86.06 (13)	O5—C17—C18	119.3 (3)
N3—V2—N4	78.04 (14)	O5—C17—C22	123.2 (3)
C7—N1—C8	117.5 (3)	C18—C17—C22	117.5 (3)
C7—N1—V1	126.5 (2)	C19—C18—C17	121.6 (4)
C8—N1—V1	115.9 (2)	C19—C18—H18	119.2
C10—N2—C9	120.3 (3)	C17—C18—H18	119.2
C10—N2—V1	129.1 (2)	C18—C19—C20	121.2 (5)
C9—N2—V1	110.6 (2)	C18—C19—H19	119.4
C23—N3—C24	119.9 (4)	C20—C19—H19	119.4
C23—N3—V2	127.4 (3)	C21—C20—C19	119.1 (4)
C24—N3—V2	112.6 (3)	C21—C20—H20	120.4
C26—N4—C25	116.5 (4)	C19—C20—H20	120.4
C26—N4—V2	127.0 (3)	C20—C21—C22	121.7 (4)
C25—N4—V2	116.5 (3)	C20—C21—H21	119.2
C1—O3—V1	130.0 (2)	C22—C21—H21	119.2
C16—O4—V1	132.7 (2)	C21—C22—C17	118.9 (4)
C17—O5—V2	131.0 (2)	C21—C22—C23	118.9 (4)
C32—O6—V2	130.3 (2)	C17—C22—C23	122.2 (4)
O3—C1—C2	119.4 (3)	N3—C23—C22	125.2 (4)
O3—C1—C6	123.9 (3)	N3—C23—H23	117.4
C2—C1—C6	116.6 (3)	C22—C23—H23	117.4
C3—C2—C1	121.4 (4)	C25—C24—N3	108.9 (4)
C3—C2—H2	119.3	C25—C24—H24A	109.9
C1—C2—H2	119.3	N3—C24—H24A	109.9
C2—C3—C4	121.2 (4)	C25—C24—H24B	109.9
C2—C3—H3	119.4	N3—C24—H24B	109.9
C4—C3—H3	119.4	H24A—C24—H24B	108.3
C5—C4—C3	118.8 (4)	N4—C25—C24	110.1 (4)
C5—C4—H4	120.6	N4—C25—H25A	109.6
C3—C4—H4	120.6	C24—C25—H25A	109.6
C4—C5—C6	121.8 (4)	N4—C25—H25B	109.6
C4—C5—H5	119.1	C24—C25—H25B	109.6
C6—C5—H5	119.1	H25A—C25—H25B	108.2
C5—C6—C1	120.2 (3)	N4—C26—C27	125.5 (4)
C5—C6—C7	118.1 (3)	N4—C26—H26	117.3
C1—C6—C7	121.5 (3)	C27—C26—H26	117.3
N1—C7—C6	125.6 (3)	C32—C27—C26	121.9 (4)
N1—C7—H7	117.2	C32—C27—C28	118.6 (4)
C6—C7—H7	117.2	C26—C27—C28	119.3 (4)
N1—C8—C9	108.0 (3)	C29—C28—C27	120.8 (5)
N1—C8—H8A	110.1	C29—C28—H28	119.6
C9—C8—H8A	110.1	C27—C28—H28	119.6
N1—C8—H8B	110.1	C28—C29—C30	120.8 (5)
C9—C8—H8B	110.1	C28—C29—H29	119.6
H8A—C8—H8B	108.4	C30—C29—H29	119.6
N2—C9—C8	106.5 (3)	C31—C30—C29	120.1 (5)
N2—C9—H9A	110.4	C31—C30—H30	119.9
C8—C9—H9A	110.4	C29—C30—H30	119.9
N2—C9—H9B	110.4	C30—C31—C32	121.8 (5)
C8—C9—H9B	110.4	C30—C31—H31	119.1
H9A—C9—H9B	108.6	C32—C31—H31	119.1
N2—C10—C11	124.8 (3)	O6—C32—C27	124.0 (4)
N2—C10—H10	117.6	O6—C32—C31	118.1 (4)
C11—C10—H10	117.6	C27—C32—C31	117.9 (4)
			
O1—V1—N1—C7	−83.8 (3)	C5—C6—C7—N1	179.1 (3)
O4—V1—N1—C7	106.3 (3)	C1—C6—C7—N1	−6.8 (5)
O3—V1—N1—C7	22.3 (3)	C7—N1—C8—C9	158.5 (3)
N2—V1—N1—C7	176.4 (3)	V1—N1—C8—C9	−21.1 (4)
O1—V1—N1—C8	95.8 (3)	C10—N2—C9—C8	129.6 (4)
O4—V1—N1—C8	−74.1 (3)	V1—N2—C9—C8	−48.7 (4)
O3—V1—N1—C8	−158.1 (3)	N1—C8—C9—N2	43.8 (4)
N2—V1—N1—C8	−4.1 (3)	C9—N2—C10—C11	−178.4 (3)
O1—V1—N2—C10	105.6 (3)	V1—N2—C10—C11	−0.5 (5)
O4—V1—N2—C10	−5.8 (3)	N2—C10—C11—C12	−175.9 (3)
O3—V1—N2—C10	−87.3 (4)	N2—C10—C11—C16	3.8 (6)
N1—V1—N2—C10	−148.8 (3)	C16—C11—C12—C13	−1.4 (6)
O1—V1—N2—C9	−76.3 (3)	C10—C11—C12—C13	178.4 (4)
O4—V1—N2—C9	172.3 (2)	C11—C12—C13—C14	0.0 (6)
O3—V1—N2—C9	90.8 (3)	C12—C13—C14—C15	1.8 (6)
N1—V1—N2—C9	29.3 (2)	C13—C14—C15—C16	−2.1 (6)
O2—V2—N3—C23	−95.3 (4)	V1—O4—C16—C15	167.7 (2)
O5—V2—N3—C23	14.7 (3)	V1—O4—C16—C11	−14.0 (5)
O6—V2—N3—C23	96.7 (4)	C14—C15—C16—O4	179.1 (3)
N4—V2—N3—C23	159.6 (4)	C14—C15—C16—C11	0.6 (5)
O2—V2—N3—C24	80.4 (3)	C12—C11—C16—O4	−177.2 (3)
O5—V2—N3—C24	−169.7 (3)	C10—C11—C16—O4	3.0 (5)
O6—V2—N3—C24	−87.6 (4)	C12—C11—C16—C15	1.1 (5)
N4—V2—N3—C24	−24.7 (3)	C10—C11—C16—C15	−178.7 (3)
O2—V2—N4—C26	85.3 (4)	V2—O5—C17—C18	−161.2 (2)
O5—V2—N4—C26	−102.8 (4)	V2—O5—C17—C22	19.7 (5)
O6—V2—N4—C26	−21.2 (4)	O5—C17—C18—C19	−179.8 (3)
N3—V2—N4—C26	−172.1 (4)	C22—C17—C18—C19	−0.7 (5)
O2—V2—N4—C25	−98.3 (4)	C17—C18—C19—C20	1.4 (6)
O5—V2—N4—C25	73.6 (5)	C18—C19—C20—C21	−0.1 (6)
O6—V2—N4—C25	155.2 (4)	C19—C20—C21—C22	−2.0 (6)
N3—V2—N4—C25	4.3 (4)	C20—C21—C22—C17	2.7 (5)
O1—V1—O3—C1	82.7 (3)	C20—C21—C22—C23	−178.1 (4)
O4—V1—O3—C1	−165.1 (3)	O5—C17—C22—C21	177.8 (3)
N1—V1—O3—C1	−24.6 (3)	C18—C17—C22—C21	−1.3 (5)
N2—V1—O3—C1	−84.2 (3)	O5—C17—C22—C23	−1.4 (5)
O1—V1—O4—C16	−89.2 (3)	C18—C17—C22—C23	179.5 (3)
O3—V1—O4—C16	163.7 (3)	C24—N3—C23—C22	179.5 (4)
N1—V1—O4—C16	80.4 (3)	V2—N3—C23—C22	−5.1 (6)
N2—V1—O4—C16	13.2 (3)	C21—C22—C23—N3	175.6 (4)
O2—V2—O5—C17	83.2 (3)	C17—C22—C23—N3	−5.2 (6)
O6—V2—O5—C17	−169.5 (3)	C23—N3—C24—C25	−143.1 (5)
N3—V2—O5—C17	−22.2 (3)	V2—N3—C24—C25	40.9 (6)
N4—V2—O5—C17	−88.6 (3)	C26—N4—C25—C24	−166.5 (5)
O2—V2—O6—C32	−82.8 (3)	V2—N4—C25—C24	16.8 (7)
O5—V2—O6—C32	166.9 (3)	N3—C24—C25—N4	−36.1 (7)
N3—V2—O6—C32	85.1 (4)	C25—N4—C26—C27	−164.8 (5)
N4—V2—O6—C32	24.2 (3)	V2—N4—C26—C27	11.6 (7)
V1—O3—C1—C2	−166.4 (2)	N4—C26—C27—C32	5.1 (7)
V1—O3—C1—C6	16.0 (5)	N4—C26—C27—C28	179.8 (5)
O3—C1—C2—C3	−179.7 (4)	C32—C27—C28—C29	0.1 (8)
C6—C1—C2—C3	−1.9 (5)	C26—C27—C28—C29	−174.8 (5)
C1—C2—C3—C4	1.1 (7)	C27—C28—C29—C30	−0.1 (9)
C2—C3—C4—C5	−0.1 (6)	C28—C29—C30—C31	−0.3 (10)
C3—C4—C5—C6	0.0 (6)	C29—C30—C31—C32	0.6 (8)
C4—C5—C6—C1	−0.9 (5)	V2—O6—C32—C27	−17.4 (5)
C4—C5—C6—C7	173.3 (3)	V2—O6—C32—C31	164.6 (3)
O3—C1—C6—C5	179.5 (3)	C26—C27—C32—O6	−3.1 (6)
C2—C1—C6—C5	1.8 (5)	C28—C27—C32—O6	−177.8 (4)
O3—C1—C6—C7	5.5 (5)	C26—C27—C32—C31	174.9 (4)
C2—C1—C6—C7	−172.1 (3)	C28—C27—C32—C31	0.1 (6)
C8—N1—C7—C6	168.6 (3)	C30—C31—C32—O6	177.6 (4)
V1—N1—C7—C6	−11.8 (5)	C30—C31—C32—C27	−0.5 (7)

### Hydrogen-bond geometry (Å, °)

**Table:**

*D*—H···*A*	*D*—H	H···*A*	*D*···*A*	*D*—H···*A*
C8—H8B···O4^i^	0.97	2.55	3.139 (3)	119
C14—H14···O3^ii^	0.93	2.56	3.364 (3)	145
C24—H24B···O1^iii^	0.97	2.34	3.178 (3)	144

Symmetry codes: (i) *x*, *y*+1, *z*; (ii) −*x*+1, *y*−1/2, −*z*; (iii) −*x*+1, *y*+1/2, −*z*+1.
